# Monocyte Distribution Width Predicts Sepsis, Respiratory Failure, and Death in COVID-19

**DOI:** 10.7759/cureus.50525

**Published:** 2023-12-14

**Authors:** Amanda Frugoli, Johnson Ong, Brittany Meyer, Bashar Khiatah, Robert Bernstein, Anthony Hernandez, Graal Diaz

**Affiliations:** 1 Graduate Medical Education/Internal Medicine, Community Memorial Hospital, Ventura, USA; 2 Pulmonary and Critical Care Medicine/Internal Medicine, Community Memorial Hospital, Ventura, USA; 3 Graduate Medical Education/Emergency Medicine, Community Memorial Hospital, Ventura, USA; 4 Graduate Medical Education, Community Memorial Health System, Ventura, USA

**Keywords:** monocyte distribution width, sars-cov-2, monocyte, biomarkers, sirs, covid-19, sepsis, mdw

## Abstract

Introduction

Sepsis is the leading cause of hospital mortality nationwide. Early recognition has been shown to improve outcomes. This research investigates the use of monocyte distribution width’s (MDW) ability to detect sepsis and clinically correlate to outcomes in COVID-19 infection.

Methods

This is a retrospective, single-center cohort study of adult patients with confirmed COVID-19 requiring hospital admission over a 14-month period (September 2020 to November 2021). MDW was evaluated as a cytomarker to predict disease severity, mortality, and determination of sepsis in patients with COVID-19. Additionally, MDW was compared to existing inflammatory markers, including procalcitonin, D-dimer, ferritin, and lactic acid.

Results

MDW was able to predict sepsis in patients with COVID-19. The average MDW was found to be significantly higher in the detection of sepsis (25.50 ± 5.93) vs. patients without (23.13 ± 4.46) (p < 0.01). MDW was able to correlate with clinical outcomes or respiratory failure/hypoxia and death. An MDW value of 24.9 was shown to be the best cut-off value in determining fatal outcomes; receiver operating characteristic curve analysis revealed an area under the curve value of 0.69 (95% CI: 0.55-0.71) with a sensitivity of 83% and specificity of 71%. A chi-square test was performed, which detected a significant association between MDW values and the final clinical outcome of COVID-19 (OR = 3.52, 95% CI: 1.78-7.11, p < 0.001). Additionally, the mean MDW of patients with hypoxia or respiratory failure was significantly higher (22 vs. 25, p < 0.1). MDW did not correlate with any of the existing inflammatory markers.

Conclusion

MDW is a novel and reliable cytomarker for identifying sepsis in patients with COVID-19 infection. High MDW values are associated with clinical outcomes of respiratory failure and death with a mortality rate or absolute risk of 25%. MDW is easily obtained from routine laboratory evaluation in the emergency room and has the potential to be a useful tool in the triage of COVID-19 patients.

## Introduction

Over the last decade, evaluation for systemic inflammatory response syndrome (SIRS) and sepsis has been a top priority for hospitals. Ongoing research has shown that early recognition of sepsis and treatments improve the nearly 30% mortality [1-3]. The most recent Surviving Sepsis Campaign in 2021 recommends using the Sequential Organ Failure Assessment (SOFA) and SIRS instead of Quick Sepsis-related Organ Failure Assessment (qSOFA) for the detection of sepsis [4]. These tools integrate the patient's physical exam findings and laboratory data to identify serious infections. Unfortunately, there are inconsistencies between the utilization of SIRS and SOFA in the literature and clinical practice, as Medicare has failed to adopt the SOFA system. There has been no single laboratory that has been able to predict sepsis.

The current COVID-19 pandemic has put increased strain on the healthcare system. Research is underway on biomarkers, calculators, and now cytomarkers for evaluating sepsis and the risk of severe disease to aid in the triage of patients. In Surviving Sepsis, the ultimate driving force for early detection of infection is timing to antibiotics, as numerous studies demonstrate that time to antibiotics impacts survival [1,2]. Despite its initial promise, there is significant variability in predicting infection, which prevented the biomarker procalcitonin from being widely accepted [5]. The 2021 Surviving Sepsis Campaign has recommended against the routine use of procalcitonin due to poor sensitivity [5,6]. Traditionally, the white blood cell (WBC) count is among the first laboratory tests available to clinicians in the ED and is used in the SIRS determination. Additionally, other components of the CBC such as bands, or immature white blood cells, are also used to clue physicians into possible infections [4]. Unfortunately, the WBC count elevation can also be nonspecific for sepsis as patients can have low, normal, or high counts and still have a serious life-threatening infection. Seymour et al. in the Third International Consensus Definitions for Sepsis and Septic Shock (Sepsis-3) identified that once patients are admitted to the hospital, the detection of infection is typically delayed by > four hours after admission [5]. For patients presenting to the hospital, delays can be > eight hours in approximately 30% of cases presenting to the ED [4,5,7]. These delays are associated with worse outcomes; therefore, there is an ongoing need for additional biomarkers that would be routinely available and reliable to help guide clinicians in evaluating patients for sepsis in the emergency room setting [7].

Beckman Coulter developed an FDA-approved cytomarker for the detection of sepsis. This cytomarker is a calculation based on monocyte distribution width (MDW) and WBCs [8,9]. It is different from biomarkers as it is based on cell size and not specific protein. The calculation uses WBC measurements known as cell morphometric parameters to characterize the cell volume variability and distribution [8,9]. The MDW calculation is as follows: MDW + WBC = area under the curve (AUC) of 0.85 [8]. This cytomarker was validated by Elliott D. Crouser et al., who conducted a multicenter, blinded, observational, prospective cohort study at three academic centers using this new biomarker to determine the diagnostic accuracy of peripheral blood MDW alone and in combination with WBC count for early sepsis detection in the emergency department [9]. They were able to identify that the MDW value of greater than 20.0 U was effective for sepsis detection [9]. Crouser et al. also found that when used in tandem with WBC, MDW was able to enhance early sepsis detection [9].

Monocytes are white blood cells that play a crucial role in the innate immune response to inflammation and infection [10]. They have the ability to differentiate into macrophages, including alveolar macrophages and dendritic cells [11]. Both differentiated cells can present antigens from digested viruses, bacteria, or other microorganisms to stimulate the adaptive immune response [11]. They have a significant role in cytokine production and may have unique responses to COVID-19. The monocyte life is about three days. A recent publication in the Lancet using this knowledge found that trending MDW can be helpful in determining clinical outcomes [6,11]. A cartooned description of the proposed pathophysiology of monocyte enlargement and cytokine production in COVID-19 is demonstrated in the Appendix.

In this retrospective study, we evaluate the new cytomarker, MDW, in the determination of sepsis in COVID-19 patients and the correlation of clinical outcomes, including hypoxia or respiratory failure and all causes of mortality.

## Materials and methods

This is a retrospective, single-center study that was conducted in a Community Hospital in Southern California. This study was approved by our Institutional Review Board (IRB). This study did not contain any personal patient information. The study population consisted of adult patients who presented to the emergency room, with positive polymerase chain reaction testing for COVID-19 and MDW measurement that required admission between September 1, 2020, and November 30, 2021. The calculation uses WBC measurements known as cell morphometric parameters to characterize the cell volume variability and distribution [8,9]. The MDW calculation is as follows: MDW + WBC = AUC of 0.85 [8]. A total of 331 were included for evaluation. No repeat values were included. Data extraction utilized the electronic medical record system using the International Classification of Diseases, Tenth Revision, Clinical Modification for the determination of SIRS, sepsis, severe sepsis, septic shock, hypoxia, and respiratory failure.

For statistical analyses, general descriptive statistics and box plots were calculated for cell population distribution parameters. The diagnostic capability was evaluated in terms of the AUC, sensitivity, specificity, positive predictive value (PPV), and negative predictive value (NPV). The scoring approach was utilized to be used to calculate confidence intervals (CIs) for sensitivity, specificity, PPV, and NPV. The Youden index will be used for the cutoff. Three approaches will be used to demonstrate the added value of MDW in comparison with WBC count. The first was the differences in AUCs. The AUC was calculated using a one-predictor variable logistic model with WBC count as the predictor and sepsis status as the response. In addition, the AUC was calculated for the two predictor variables logistic model with both WBC count and MDW as predictors and sepsis as a response. The comparison between the AUC from the two models (WBC count vs. WBC count þ MDW) along with their CIs were calculated. The second was the Cochran-Mantel-Haenszel (CMH) approach. Both WBC count and MDW were dichotomized to 0 and 1 based on their values falling into the normal or abnormal category. Comparing the binary threshold variable indicator indicating MDW values greater than or less than 24.9 against survival status, a chi-square test was performed, which detected a significant association between MDW values and final clinical outcomes of COVID-19. The following statistical analyses were applied through the use of open-source R statistical software packages (https://www.r-project.org) to correlate MDW values with routine laboratory parameters and final outcomes.

## Results

The mean age of this population (N = 331) was 64.25 ± 16.78 years old. There were 168 (50.76%) male and 163 (49.24%) female patients. Table 1 summarizes the demographics of the study population.

**Table 1:** Descriptive characteristics of the population ICU: intensive care unit; MDW: monocyte distribution width.

Variable	N	Percent (%)
Age group		
18-44	48	14.50%
45-54	40	12.08%
55-64	65	19.64%
65-74	69	20.85%
75+	109	32.93%
Gender		
Male	168	50.76%
Ethnicity		
White	126	38.07%
Asian	9	2.72%
Black/African American	5	1.51%
Hispanic/Latino	191	57.70%
MDW		
Greater than 20 (>20)	247	74.62%
Less than 20 (<20)	84	25.38%
ICU hospitalization		
Yes	91	27.49%
No	240	72.51%
Outcomes		
Discharged (survivors)	284	85.80%
Expired (death)	47	14.20%
Sepsis		
Yes	71	21.45%
No	260	78.55%
Sepsis severity		
No sepsis	260	78.55%
SIRS (systemic inflammatory response syndrome)	3	0.91%
Sepsis	8	2.42%
Severe sepsis	36	10.88%
Septic shock	24	7.25%

MDW as well as inflammatory biomarker values, such as ferritin, D-dimer, procalcitonin, and lactic acid, were collected and compared against any level of sepsis (SIRS, sepsis, severe sepsis, or septic shock). Average MDW was found to be significantly higher in patients with sepsis (25.50 ± 5.93) vs. patients without (23.13 ± 4.46) (p < 0.01); average D-dimer values were found to be significantly higher in patients with sepsis (7560.11 ± 17698.53) vs. patients without (1933.25 ± 3725.41) (p = 0.02); average procalcitonin values were found to be significantly higher in patients with sepsis (2.91 ± 6.10) vs. patients without (0.95 ± 4.47) (p = 0.02); average lactic acid values were found to be significantly higher in patients with sepsis (2.33 ± 1.88) vs. patients without (1.64 ± 0.84) (p < 0.01) (Table 2).

**Table 2:** Comparison table of inflammatory markers including MDW in the detection of sepsis Average MDW was found to be significantly higher in patients with sepsis (25.50 ± 5.93) vs. patients without (23.13 ± 4.46) (p < 0.01). MDW: monocyte distribution width.

	Sepsis	No sepsis		
Variable	Mean +/- SD	Mean +/- SD	t (95% CI)	P-value
MDW	25.50 +/- 5.93	23.13 +/- 4.46	-3.13 (-3.87, -0.86)	<0.01
Ferritin	858.47 +/- 1164.07	638.80 +/- 826.44	-1.17 (-539.62, 154.29)	0.25
D-dimer	7560.11 +/- 17698.53	1933.25 +/- 3725.41	-2.38 (-10353.05, -900.67)	0.02
Procalcitonin	2.91 +/- 6.10	0.95 +/- 4.41	-2.28 (-3.68, -0.25)	0.02
Lactic acid	2.33 +/- 1.88	1.64 +/- 0.84	-2.91 (-1.16, -0.22)	<0.01

Average MDW was found to be significantly higher in patients with sepsis (25.50 ± 5.93) vs. patients without (23.13 ± 4.46) (p < 0.01).

Results demonstrating average MDW were found to be significantly higher in patients with sepsis (25.50 ± 5.93) vs. patients without (23.13 ± 4.46) (p < 0.01).

In this study cohort, there were 284 (85.54%) favorable cases where patients were discharged to home or other care settings, and 47 (14.16%) had fatal outcomes. Applying the Mann-Whitney test for independent values, we found a significant correlation (p < 0.001) between the MDW detected in each patient (n = 331), and the final clinical outcome (survival/discharge vs. expiration). The median MDW value was about 24.9 between the 47 patients with death, which could be indicative of a prognostic threshold. Receiver operating characteristic (ROC) curve analysis is a statistical tool based on the notions of specificity and sensitivity, used for assessing diagnostic tests and predictive models. We utilized this approach to determine the best MDW cut-off to assess the probability of fatal outcomes. We did not have repeat MDW variables and only presenting MDW was utilized. We identified an MDW value of 24.9 as the best cut-off to assess the probability of fatal outcomes during the disease course in our cohort of COVID-19 patients. This analysis provided an AUC value of 0.69 (95% CI: 0.55-0.71; sensitivity: 0.83; specificity: 0.71). Among 118 patients with an MDW value of >24.9, 29 cases had fatal outcomes, meaning that high MDW values are associated with a mortality rate or absolute risk of about 25%. A total of 18 out of 213 patients (9%) died while presenting MDW values lower than 24.9 (NPV = 0.91). Comparing the binary threshold variable indicator indicating MDW values greater than or less than 24.9 against survival status, a chi-square test was performed, which detected a significant association between MDW values and final clinical outcomes of the COVID-19 patient (OR = 3.52, 95% CI: 1.78-7.11, p < 0.001), which indicates that MDW values are associated with clinical outcomes in COVID-19 patients.

Respiratory failure and hypoxia were determined on ICD billing codes. A two-tailed t-test was completed comparing the average MDW between COVID-19 patients with respiratory failure. The MDW was statistically higher in patients with respiratory failure (24.9 vs. 21.9; p < 0.01).

## Discussion

This research demonstrates that MDW is a reliable and easily exploitable cytomarker that can be utilized in patients with COVID-19 to detect sepsis and correlate with clinical outcomes of hypoxia and respiratory failure. Given much is still being discovered about this pandemic and the triage of patients, it is important to recognize the niche of this novel marker's potential in evaluating patients. MDW is a marker of the immune response to inflammation and unlike other biomarkers, it is calculable from the WBC count [8]. This is unique since patients with COVID-19 can present with relative leukopenia; it is important to recognize that the monocyte count distribution width was still able to identify ill patients and correlate with clinical outcomes in this study. Dedicated research on MDW in patients with specific illnesses that can affect monocyte production and maturation will need to be completed to determine utilization.

MDW correlates with clinical outcomes of survival

The biggest discovery identified is that an MDW value of 24.9 (25) is able to correlate with fatal outcomes. In fact, an MDW of greater than 24.9 had an absolute risk of 25%. Patients with a lower MDW had a negative predictive value of 0.91. When we compared the binary thresholds of MDW, they correlated to final outcomes with an odds ratio of 3.5. This is similar to Riva et al., except we are able to demonstrate a slightly lower MDW value of 25 (24.9) as a powerful predictor [12,13]. Our research also supports Lippi et al.’s pooled analysis that illustrated that MDW at hospital admission is higher in subjects with COVID-19 [14]. Furthermore, our research adds diagnostics values to clinical correlations using a larger population.

MDW is able to detect hypoxia and respiratory failure

COVID-19 patients with hypoxia and respiratory failure had a significantly higher average MDW value. The MDW value for respiratory failure matched the MDW prediction for death. Since respiratory failure is one of the biggest clinical outcomes for COVID-19, it is not surprising that these are correlated. It is important to note that the MDW was helpful for risk stratification. Our data support Hossain et al., who found that there is a meaningful difference in MDW in patients with hypoxic respiratory failure [15]. Our data utilized the International Classification of Diseases (ICD) codes in the determination of hypoxia and respiratory failure, and we were able to demonstrate that patients with respiratory problems had a significantly higher MDW of 24.9 compared to those without respiratory failure (24.9 vs. 21.9; p < 0.01).

MDW is able to detect sepsis in COVID-19

We are also able to illustrate that MDW was able to detect sepsis in patients with COVID-19. We compared MDW as well as the inflammatory biomarker ferritin, D-dimer, procalcitonin, and lactic acid and compared against whether the patient had any level of sepsis (SIRS, sepsis, severe sepsis, or septic shock). The average MDW was found to be significantly higher in patients with sepsis (25.50 ± 5.93) vs. patients without (23.13 ± 4.46) (p < 0.01).

Surprisingly, MDW did not correlate with inflammatory markers, D-dimer, procalcitonin, lactic acid, or ferritin. This is dissimilar to Riva et al., where MDW correlated with laboratory values of inflammation, including C-reactive protein (CRP), ferritin, and fibrinogen [12,13]. These are not routinely utilized inflammatory markers and may represent an area of further research. It is possible that CRP, ferritin, and fibrinogen may be more specific to COVID-19. These markers were not available for comparison in our study. However, procalcitonin, lactic acid, and D-dimer are routinely used in the emergency department for the determination of inflammatory response to COVID-19. It is possible that this is directly linked to the pathophysiology of monocytes and their role in COVID-19 and cytokine production [16]. MDW may have a specific niche for COVID-19, especially since MDW did not correlate with other biomarkers. Our study differs from Ennio Polilli et al., as we found the MDW did not correlate with procalcitonin in the detection of sepsis [17]. It is possible that MDW is able to predict a new kind of sepsis or viral sepsis. This is also similar to Piva et al., who found MDW increase is higher in patients with sepsis, and the MDW increase was not affected by the etiology of sepsis even in patients with COVID-19 [6].

Strengths and weakness

Due to the retrospective nature of this study design, we are unable to control for confounding variables. To mitigate this, we created a very specific and homogenous population in hopes of making the evidence meaningful and applicable. We hope to repeat this study and compare MDW's ability to predict sepsis in patients with and without COVID-19. Additionally, our homogenous population of admitted patients may have allowed for selection bias for sicker patients with increased risk for sepsis or death. Currently, our hospital and staff primarily use Sepsis-2 criteria for the identification of sepsis because it correlates to ICD coding and billing. However, future studies can evaluate MDW with SOFA criteria from Sepsis-3. Our methods are similar to the recent study published in Nature by Riva et al. for mortality prediction using MDW [12,13]. This study is expanded to compare MDW against existing biomarkers to compare sepsis, respiratory failure, and death, and also has a larger sample size.

This study could have been stronger if we had been able to further differentiate COVID-19 from secondary bacterial infections using microbiotic data from sputum cultures and blood cultures. In this study, the MDW was not evaluated to determine if sepsis was related to viral infection with COVID-19 or other co-occurring bacterial infections. In the setting of COVID-19, having a marker for bacterial infections would be clinically useful for antimicrobial stewardship.

## Conclusions

MDW is a novel and reliable cytomarker for identifying sepsis in patients with COVID-19 infection. High MDW values are associated with clinical outcomes of respiratory failure and death with a mortality rate or absolute risk of 25%. MDW is easily obtained from routine laboratory evaluation in the emergency room and has the potential to be a useful tool in the triage of COVID-19 patients.

## Figures and Tables

**Figure 1:**
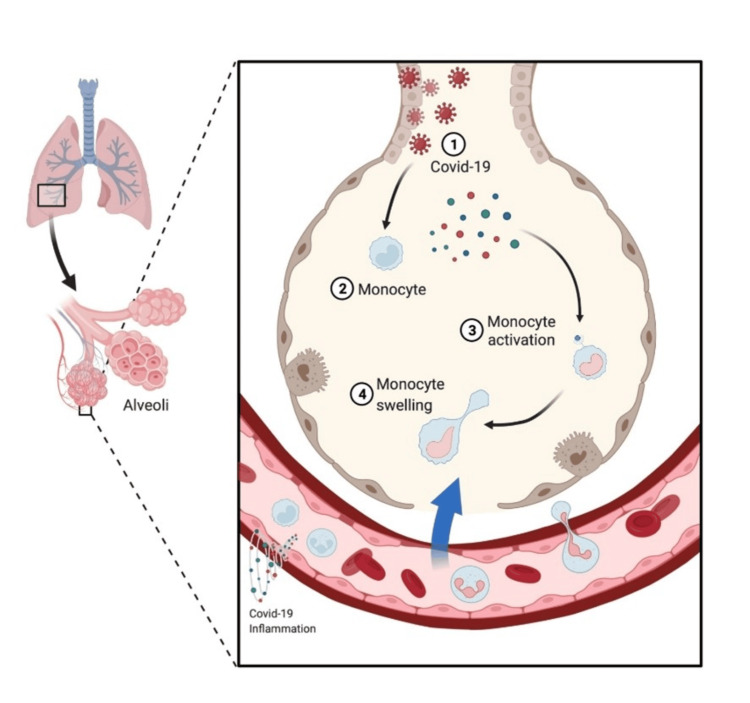
Cartooned description of proposed pathophysiology of monocyte enlargement and subsequent cytokine production in COVID-19 Image made with the use of BioRender. Permission and publication licensing obtained for use.
